# Morphology of Chinese Chive and Onion (*Allium*; Amaryllidaceae) Crop Wild Relatives: Taxonomical Relations and Implications

**DOI:** 10.3390/plants15020192

**Published:** 2026-01-07

**Authors:** Min Su Jo, Ji Eun Kim, Ye Rin Chu, Gyu Young Chung, Chae Sun Na

**Affiliations:** 1 Division of Wild Plant and Seeds, Baekdudaegan National Arboretum, Bonghwa 36209, Republic of Korea; jms991@koagi.or.kr (M.S.J.); jekim803@koagi.or.kr (J.E.K.); yerinchu@koagi.or.kr (Y.R.C.); 2 Department of Forest Sciences, Kyeongkuk National University, Andong 36729, Republic of Korea; gychung@andong.ac.kr

**Keywords:** *Allium*, crop wild relative, seed, micro-morphology, phylogeny, taxonomy

## Abstract

The genus *Allium* L. includes economically significant crops such as Chinese chives (*Allium tuberosum* Rottler ex Spreng.) and onions (*Allium cepa* L.), and is utilized in diverse agricultural applications, with numerous cultivars developed to date. However, these cultivars are facing a reduction in genetic diversity, raising concerns regarding their long-term sustainability. Crop wild relatives (CWRs), which possess a wide range of genetic traits, have recently gained attention as important genetic resources and priorities for conservation. In this study, the taxonomy of *Allium* species distributed in Korea is assessed using morphological characteristics. Two types of morphological analyses were conducted: macro-morphological traits were examined using stereomicroscopy and multi-spectral image analyses, while micro-morphological traits were analyzed using scanning electron microscopy. We detected significant interspecific and intraspecific variation in macro-morphological traits. Among the micro-morphological features, the seed outline on the *x*-axis and structural patterns of the testa and periclinal walls were identified as reliable diagnostic characters for subgenus classification. Moreover, micro-morphological evidence contributed to inferences about evolutionary trends within the genus *Allium*. Based on phylogenetic relationships between wild and cultivated taxa, we propose an updated framework for the CWR inventory of *Allium*.

## 1. Introduction

The genus *Allium* L.(Amaryllidaceae) includes over 1000 species worldwide, making it one of the largest genera within monocotyledons [1]. Historically, members of this genus, such as onion (*A. cepa* L.), scallion (*A. fistulosum* L.), and garlic (*A. sativum* L.) have been widely cultivated and utilized as food crops [2]. Extensive breeding efforts have led to the development of numerous cultivars. Furthermore, the taxonomic and morphological characteristics of *Allium* species have been the subject of considerable research. Traditionally, *Allium* was classified within the family Liliaceae; however, based on molecular phylogenetic evidence, the APG (Angiosperm Phylogeny Group) system has reclassified *Allium* and related genera under the family Amaryllidaceae [3,4].

Approximately 21 *Allium* species have been reported on the Korean Peninsula [5]. Among these, *A. longistylum* Baker is listed as Least Concern, while *A. dumebuchum* H.J.Choi and *A. microdictyon* Prokh. are categorized as Near Threatened in the Korean Red List [6]. These taxa and other wild relatives of cultivated crops (crop wild relatives; CWRs) are experiencing population declines due to habitat loss driven by agricultural expansion and hybridization with domesticated varieties [7]. To ensure the conservation of these vulnerable taxa and their associated genetic resources, detailed morphological and phylogenetic studies are essential.

Crop wild relatives (CWRs), the wild plants and close relatives of cultivated crops, are a rich source of readily accessible genetic material for crop improvement and are considered invaluable for modern plant breeding [8,9]. All domesticated crops used by humans today have originated from CWRs. For instance, *Oryza barthii* A.chev. is the wild ancestor of rice [10] and *Solanum pennellii* Correll is a recognized origin of tomato [11]. Although original wild ancestors may have become extinct or undergone significant genomic changes due to polyploidization or other evolutionary processes, closely related CWRs continue to provide essential genetic resources [12]. Climate change and the resulting threat to global food security have led to increased research interest in CWRs [13,14]. In Europe, international initiatives, such as the CROPTRUST, are actively focused on collection, inventory development, and conservation.

The morphological classification of the genus *Allium* has been conducted using a wide range of plant materials. Jang et al. [15] suggested that, based on floral morphological characteristics, ovary shape, perianth shape, and the inner tepal apex are useful at the subgenus level from a phylogenetic perspective. However, they also noted that quantitative traits such as tepal size exhibit intraspecific variation, making it difficult to identify *Allium* species solely on that basis. Other studies have examined the pollen of *Allium*, confirming that pollen longitude axis, sulcus length, and exine ornamentation are informative characters at the subgenus level or section level [16,17]. Furthermore, even in the bulb tunic has been studied for its anatomical characteristics, and it has been proposed that the shape and type of subepidermal cells, the number, distribution, type, and size of crystals, the tracheid type, and the presence of bulbils can serve as diagnostic traits for distinguishing subgenus and section level [18].

Seeds of *Allium* exhibit substantial morphological similarity, rendering species-level identification challenging [19]. As a result, the taxonomic boundaries within the genus are continuously revised. Recent research has improved our understanding of *Allium* taxonomy in Korea. For example, based on macro-morphological traits, Choi and Oh [20] recognized *A. sacculiferum* Maxim. and *A. thunbergii* G.Don. as distinct species; however, they also reported considerable intraspecific morphological variation. Shukherdorj et al. [21] interpreted this variation as a result of polyploidy within *A. thunbergii*, leading to the taxonomic merging of *A. sacculiferum* into *A. thunbergii*. Similarly, *A. ulleungense* H.J.Choi & N.Friesen, previously considered a regional variant of *A. microdictyon* or conspecific with *A. ochotense*, was reclassified as a Korean endemic species based on molecular phylogenetic analyses using ITS and chloroplast DNA sequences [22]. *A. dumebuchum*, historically treated as *A. senescens* L. in Korea, was also recently recognized as a distinct species through combined cpDNA and morphological analyses [23].

In traditional morphological studies, traits such as color and shape are determined based on the subjective judgment of the researcher. Recently, there is a trend toward the use of quantitative indicators (e.g., parameters expressed in numbers or coordinates according to wavelength and image analysis techniques). Such quantitative traits have been applied in paleontology, oceanography, and other fields [24,25]. In plant morphology, recent systematic classification studies have used leaf traits [26,27] and the shapes of other organs, such as flowers and seeds [28,29]. However, studies using seeds of the genus *Allium* are lacking.

Micro-morphological seed traits are important for species classification. In particular, the micromorphology of seeds is not highly sensitive to environmental variables, and many studies have confirmed its usefulness above a certain rank. In the genus *Allium*, the shapes of the protrusion and scutellum indicate the evolutionary development stage [30], and the taxonomic value of the shape and curvature of the scutellum has been established [1]. However, research has focused largely on the outer layer of the seed coat, and overall analyses of the seed coat on the seed cross-section are still insufficient, despite potential implications for classifying taxa [31,32,33]. The genus Allium comprises approximately 15 subgenera and 80 sections worldwide [1]. In this study, we established a classification based on morphological characteristics using seeds from 11 taxa and 3 cultivated varieties of *Allium* distributed in Korea, which belong to 4 subgenera and 5 sections. We confirmed the species relationships using the external shape and micro-morphology and determined the taxonomic value of these characteristics. In addition, we inferred the evolutionary trends of each character based on integrative analyses with existing molecular phylogenetic data. Finally, we propose a list of existing inventories of taxa as CWR. The terminology used in this study corresponds with Murley [34] and Harris & Harris [35].

## 2. Results

### 2.1. External Morphology

**Figure 1:**
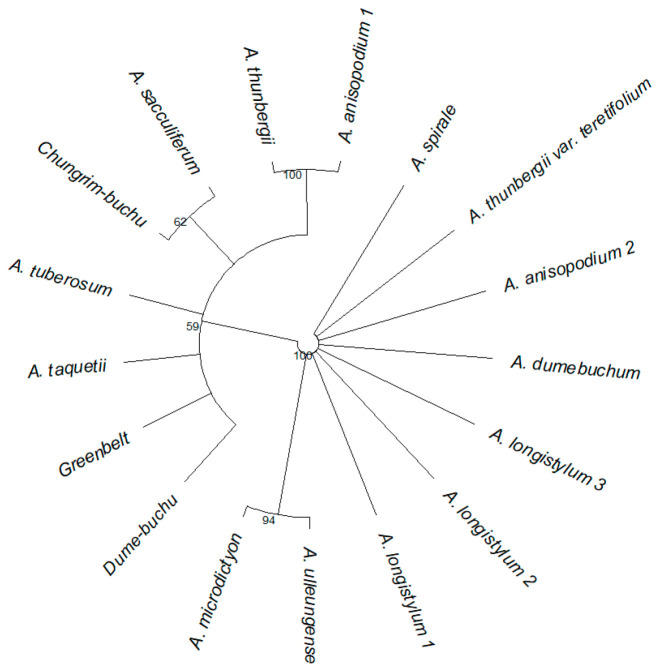
*Allium* seed external morphological similarity dendrogram between taxa. The tree was calculated by NJ and bootstrap value = 1000. External morphological varieties were measured Red, Green, Blue, Length, Width, Height, Beta shape a, Beta shape b, Ellipse Compactness, Circle Compactness, Area, Width/Area, Width/Length, Rectangularity, Region Horizontal Length Mean, Region Vertical Length Mean, Skew x, Skew y, Phi Rad Symmetry Stat Mean, Phi Rad Symmetry Stat Max, Kurt x, Kurt y, Moment x, Moment y, Moment Y Ratio, Perimeter, Phi Rad Statistics Average, Phi Rad Statistics Min, Phi Rad Statistics Max and Pointness.

In total, 30 morphological characters were evaluated (Appendix A). In each character, evaluated with other taxon, some of taxon differed significantly (Figure 1). In particular, *A. anisopodium* Ledeb., *A. microdictyon*, and *A. ulleungense* differed from other taxa in the most traits. In the case of cultivated varieties, *Greenbelt* and *Chungrim-bucu* shared common characters with *A. longistylum* and *A. thunbergii* and showed substantial divergence in characters from the CWR, *A. tuberosum* Rottler ex Spreng. *Dume-buchu* also shared common characters with *A. thunbergii* and *A. dumebuchum*.

#### 2.1.1. Interspecific Variation

There were significant differences in many traits within groups of *A. anisopodium*; accordingly, we performed additional comparisons between *A. longistylum* and *A. anisopodium* populations (Table 1). In particular, we analyzed the average differences in trait values between three groups of *A. longistylum* and two groups of *A. anisopodium* collected from populations in different regions. Differences between groups were observed in most of the 30 variables. The average values for Height, Beta shape a and b, Rectangularity, Skew x and y, Kurt x and y, Phi Rad Statistic Average, Pointness, Ellipse and Circle Compactness, and Moment y Ratio were similar across groups.

#### 2.1.2. Seed Outline Analysis

**Figure 2:**
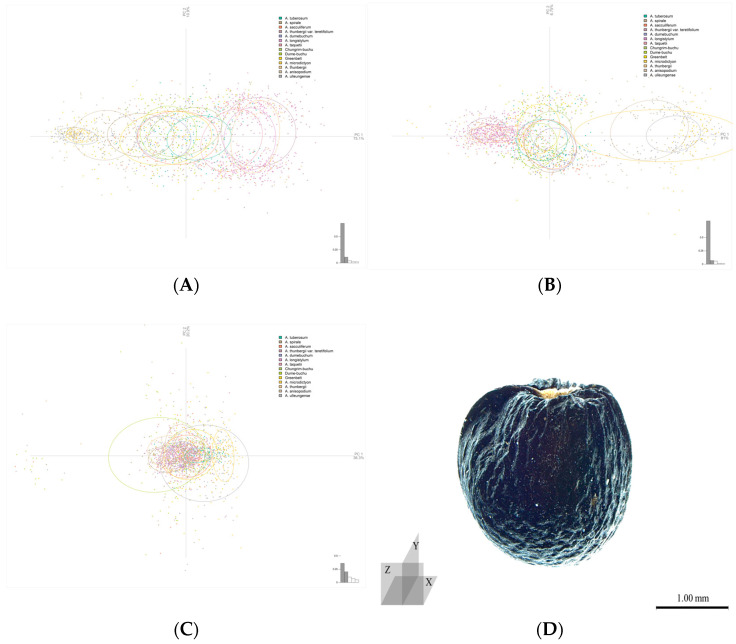
Principal component analysis (PCA) results for each axis and the cut direction of each axis. (**A**–**C**). summarize results for the *x*-, *y*-, and *z*-axes. (**D**). Cut direction of *A. microdictyon*. The ellipses for each axis represent 70% of the range of the entire population based on the center point of one taxon.

A principal component analysis (PCA) using the coordinate values of all outlines confirmed the accuracy of taxon classification on the *x*- and *y*-axes, excluding the *z*-axis (Figure 2, Table 2). On the *x*- and *y*-axes, *A. anisopodium*, *A. microdictyon*, and *A. ulleungense* were clustered, and *A. thunbergii*, *A. thunbergii* var. *teretifolium,* and *A. longistylum* had similar outlines. Furthermore, the outline of *A. microdictyon* on the *y*-axis had greater variation than those of other taxa. Along the *z*-axis, all taxa formed a single group without any special distinction. The PCA confirmed that the three cultivars had very similar outlines to those of the CWRs *A. tuberosum* and *A. dumebuchum*.

### 2.2. Micromorphological Analysis

**Figure 3:**
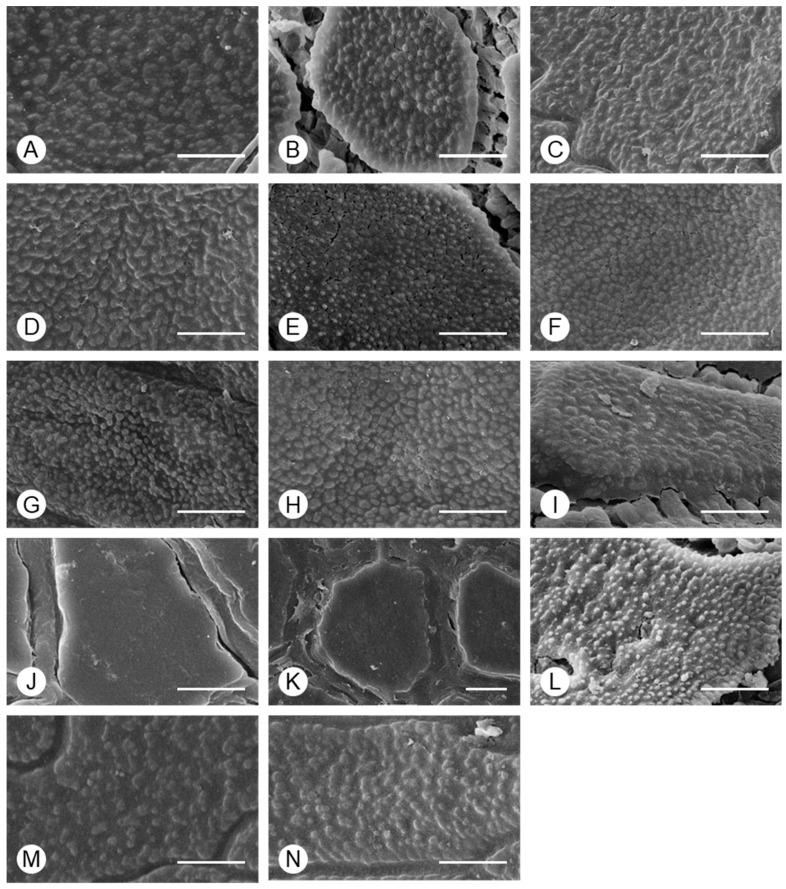
SEM-based characterization of the periclinal wall of *Allium* seeds. (**A**) *A. tuberosum*, (**B**) *A. spirale*, (**C**) *A. sacculiferum*, (**D**) *A. thunbergii* var. *teretifolium*, (**E**) *A. dumebuchum*, (**F**) *A. longistylum*, (**G**) *A. taquetii*, (**H**) *A. thunbergii*, (**I**) *A. anisopodium*, (**J**) *A. ulleungense*, (**K**) *A. microdictyon*, (**L**) *Dume-buchu*, (**M**) *Greenbelt*, (**N**) *Chungrim-buchu*. Scale bars represent 10 μm.

In analyses of the micro-morphology of *Allium* seeds, the surface of the periclinal wall was classified as granulate in all taxa, except *A. microdictyon* and *A. ulleungense* (Figure 3). Although there were significant differences between some species in the size of granulate, the range was large, even within individuals (Table 3). The granulates on the periclinal wall were density distributed, such that the base was not visible, with fusion to nearby granulates in some cases.

**Figure 4:**
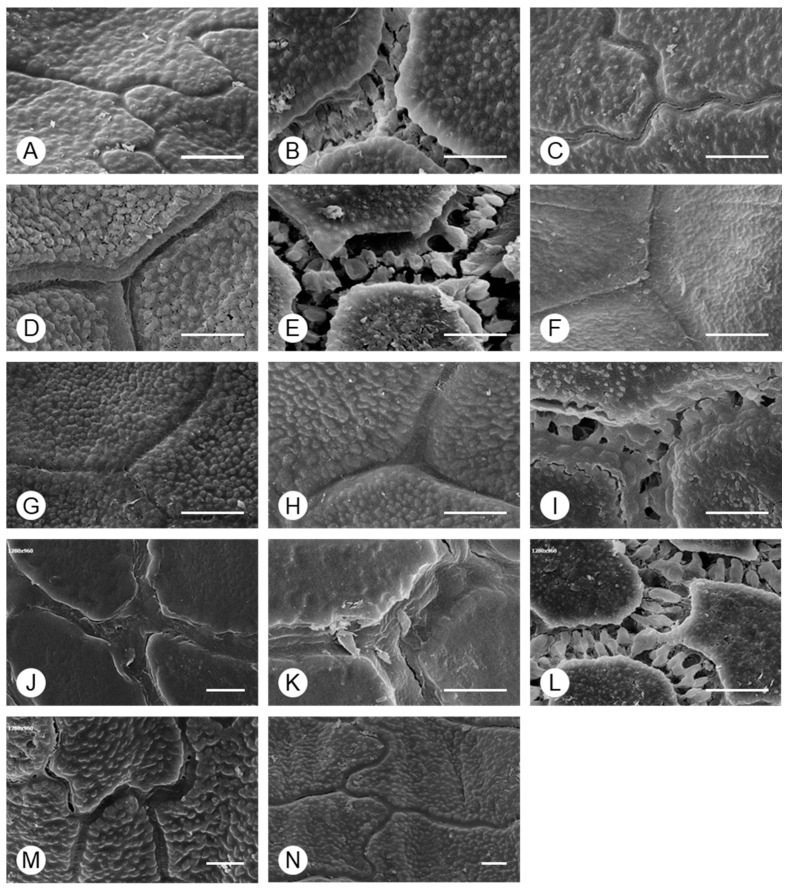
Morphology of the anticlinal wall boundary of *Allium* seeds determined using SEM. (**A**) *A. tuberosum*, (**B**) *A. spirale*, (**C**) *A. sacculiferum*, (**D**) *A. thunbergii* var. *teretifolium*, (**E**) *A. dumebuchum*, (**F**) *A. longistylum*, (**G**) *A. taquetii*, (**H**) *A. thunbergii*, (**I**) *A. anisopodium*, (**J**) *A. ulleungense*, (**K**) *A. microdictyon*, (**L**) *Dume-buchu*, (**M**) *Greenbelt*, (**N**) *Chungrim-buchu*. Scale bars represent 10 μm.

**Figure 5:**
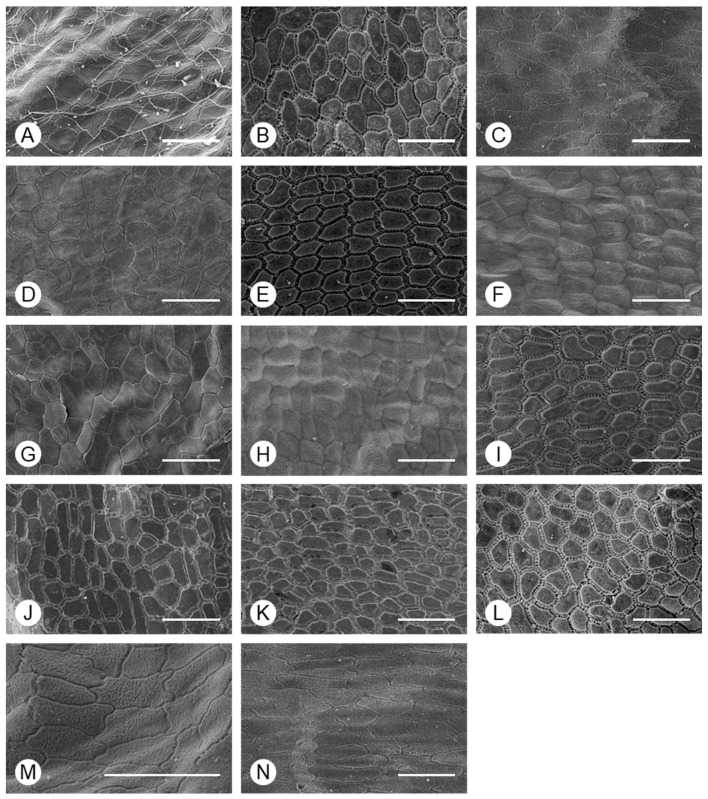
Morphology of the periclinal wall of *Allium* seeds, determined using SEM. (**A**) *A. tuberosum*, (**B**) *A. spirale*, (**C**) *A. sacculiferum*, (**D**) *A. thunbergii* var. *teretifolium*, (**E**) *A. dumebuchum*, (**F**) *A. longistylum*, (**G**) *A. taquetii*, (**H**) *A. thunbergii*, (**I**) *A. anisopodium*, (**J**) *A. ulleungense*, (**K**) *A. microdictyon*, (**L**) *Dume-buchu*, (**M**) *Greenbelt*, (**N**) *Chungrim-buchu*. Scale bars represent 100 μm.

Two types of anticlinal wall shapes were observed (Figure 4): (I) irregularly curved (*A. tuberosum* and *A. sacculiferum*) and (II) straight. In addition, the thickness of the anticlinal walls and type of connections between cells differed among taxa and could be divided into three types: (i) thick anticlinal walls but cells connected in a linear form (*A. microdictyon* and *A. ulleungense*), (ii) cells connected through pilates of anticlinal walls (*A. dumebuchum* and *Dume-buchu*), and (iii) thin anticlinal walls and cells connected in a linear form. To confirm the types of anticlinal walls in each taxon, the parts where individual cells were connected and where the anticlinal walls between cells had separated due to partial destruction were observed (Figure 5). In taxa with curved anticlinal walls, the overall form of the seed coat cells was elliptical with a long equatorial axis. When cell boundaries were parallel to the major axis, they were straight, with curves forming only at the ends. By contrast, in taxa with straight boundaries, the shape was nearly hexagonal.

**Figure 6:**
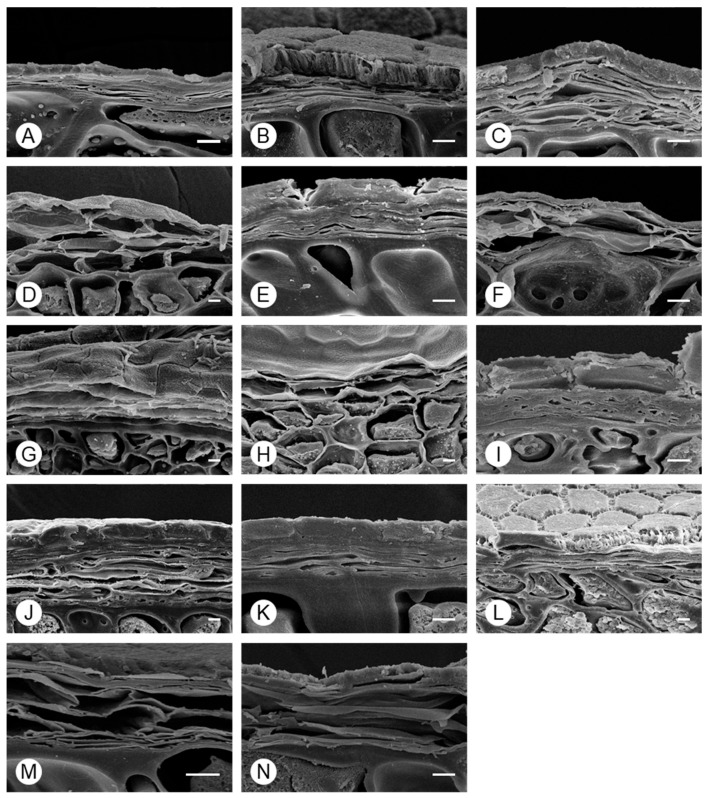
Morphology of *Allium* seed coats, determined using SEM. (**A**) *A. tuberosum*, (**B**) *A. spirale*, (**C**) *A. sacculiferum*, (**D**) *A. thunbergii* var. *teretifolium*, (**E**) *A. dumebuchum*, (**F**) *A. longistylum*, (**G**) *A. taquetii*, (**H**) *A. thunbergii*, (**I**) *A. anisopodium*, (**J**) *A. ulleungense*, (**K**) *A. microdictyon* (**L**) *Dume-buchu*, (**M**) *Greenbelt*, (**N**) *Chungrim-buchu*. Scale bars represent 10 μm.

Scanning electron microscopy of cross-section of seeds in the genus *Allium* showed that the exotesta had a structurally complex shape and was composed of a relatively hard tissue in all taxa (Figure 6). Apart from for the exotesta, the cell boundaries consisted of a simple membrane. Owing to changes during preprocessing, only the simple structure was confirmed. The outer layer of the outer seed coat was composed of a single layer of cells and had a hard structure; as this structure was maintained during preprocessing, it could be compared among taxa. The outer layer was thicker in *A. microdictyon*, *A. ulleungense*, *A. dumebuchum*, and *Dume-buchu* than in other taxa.

### 2.3. Phylogenetic Analysis

#### 2.3.1. Phylogenetic Analysis of the Outline

**Figure 7:**
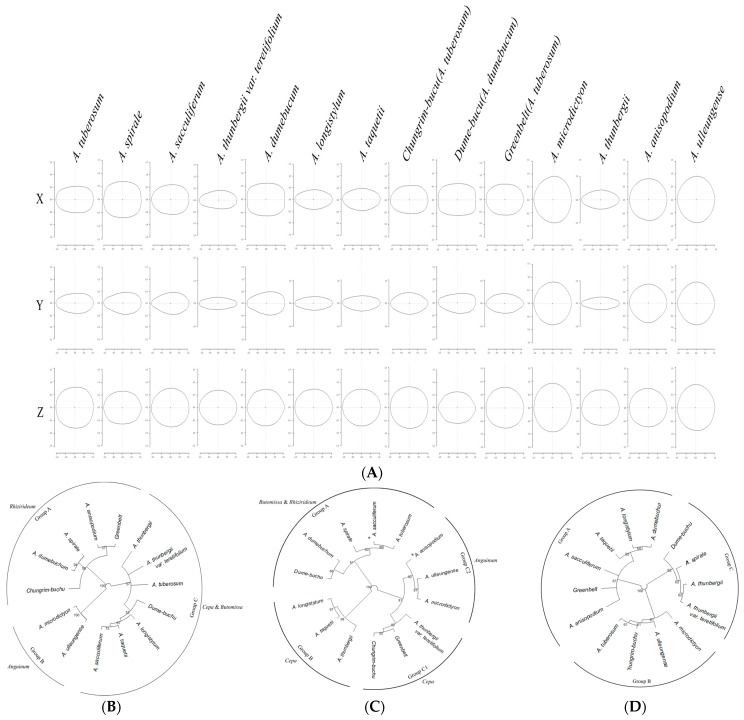
Average form on each axis for each taxon and phylogenetic analysis based on the average form for each axis. The average form was drawn based on the eFourier transformation. (**A**) Average outline of each taxon, (**B**) phylogeny based on the seed *x*-axis outline, (**C**) phylogeny based on the seed *y*-axis outline, (**D**) phylogeny based on the seed *z*-axis outline. For each phylogeny, the optimal groups were distinguished and described according to the bootstrap value (based on 1000 replicates).

Each taxon was classified based on the outline shape on the *x*-, *y*-, and *z*-axes of *Allium* seeds (Figure 7). Along the *x*-axis, samples were divided into three groups, Group A consisting of subg. *Rhizirideum*, Group B divided into subg. *Anguinum*, and Group C divided into subg. *Cepa* & *Butomissa*. The subgenera were clearly distinguished based on the *x*-axis. For the *y*-axis, the classification results were less clear than those for the *x*-axis; Subg. *Cepa* could be largely divided into two groups. For the *z*-axis, all taxa showed similar morphological characteristics and were divided into three groups, without any distinction between subgenera.

#### 2.3.2. Phylogenetic Analysis

**Figure 8:**
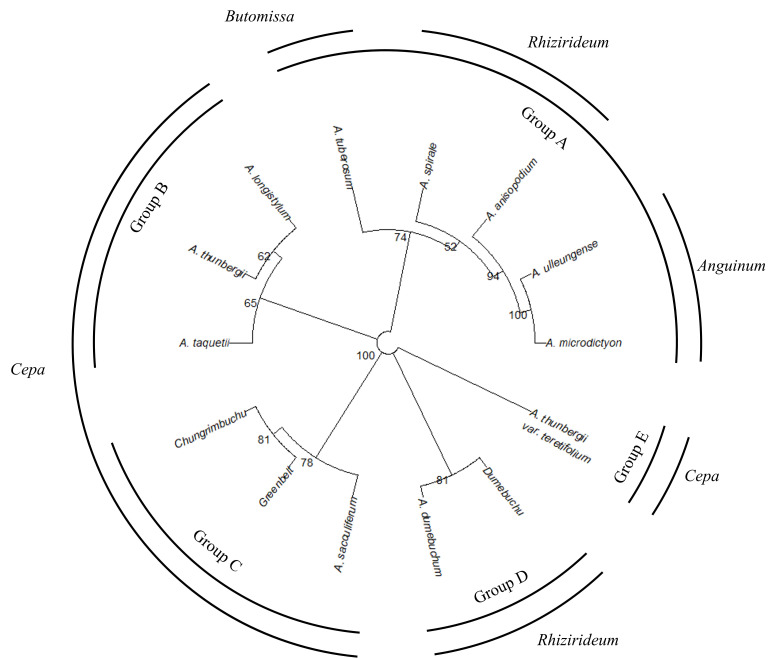
Comprehensive phylogenetic analysis of *Allium* based on all characters. The phylogenies were calculated through the optimal distance and clustering method based on the Factor Analysis Mixed Data results, and the optimal tree was selected with a Bootstrap value of 1000. The tree was divided into five groups.

A comprehensive phylogenetic analysis was performed based on all studied characters (Figure 8). Taxa were divided into five groups. Subgenera *Anguinum*, *Rhizirideum*, and *Butomissa* formed one group (Group A in Figure 8). Subgenus *Cepa* was divided into three groups (Groups B, C, and E). *A. thunbergii* var. *teretifolium* formed an independent group. Additionally, *A. dumebuchum* formed an independent group, distinct from other taxa in subg. *Rhizirideum*. The cultivar *Dumebuchu* formed a group with *A. dumebuchum*, its CWR, while *Greenbelt* and *Chungrim-buchu* formed a group with *A. sacculiferum*.

## 3. Discussion

Analyses of the external, internal, and micromorphological characteristics of seeds revealed that the seed outline is a useful characteristic for identification at the subgenus level. However, other external morphological characteristics showed differences between populations within the same taxonomic group. Characteristics such as width and length show variation depending on genetic characteristics, even within very small populations [36]. Although these genetic characteristics provide a basis for distinguishing taxa [37], the traits are also determined by the environmental characteristics of the native region [38]. Accordingly, studies of the external morphological characteristics of seeds should consider environmental variables, and inter-intra population analyses are needed to understand trait variation and infer relationships.

Seed coat color and external morphology are often similar in different taxa, limiting their use for accurate classification. To ensure objectivity, color is measured using wavelengths [39,40], and outlines are quantitatively assessed using Fourier transform methods. *Allium* seeds are commonly (almost) black, with an oval-flattened/angular shape [41]. Our quantitative trait analyses indicated that the seed color is moderate olive green–grayish brown, and the seed outline is elliptical–spherical based on the *z*-axis. There were no significant differences in seed color between taxa; however, RGB values were correlated. There were no significant differences among populations within taxa; however, there was considerable variation within populations (Appendix A). Recent studies have utilized color changes during seed harvesting and storage as indicators of seed viability [42]. Given that seed color varies with viability, comprehensive RGB analyses, rather than individual color channels, are required for *Allium* species. Such analyses are expected to reveal significant interspecific differences in RGB values.

In the seed outline analysis, the *x*-axis was useful for classification. Fourier transform is mainly applied to leaves [26,43] and has rarely been applied to seeds. Nevertheless, our findings indicate that the *Allium* seed outline may be effective for species identification using machine learning techniques. Various machine learning techniques have been used for species classification, including studies based on outlines [44] or comprehensive traits, including outlines, through multispectral imaging [45]. These methods are expected to improve the accuracy of species classification and may be applicable to similar traits within the genus *Allium*.

Micromorphological traits of seeds have been used in phylogenetic analyses [30]. The results of this study indicate that the presence or absence of granules on the periclinal wall and shape of the anticlinal wall are useful for species classification at the subgenus level. These findings support the classification results based on various types of traits in previous studies of the genus *Allium*. However, the cross-section of the testa of *Allium* seeds has not been studied. In this study, the exotesta was thick in *Anguinum* and *Rhizirideum* and was thin in *Cepa* and *Butomissa* (Figure 6), indicating its value for morphological classification at the subgenus level. Previous studies have reported that the tegmen collapses or fuses with the endotesta during seed development [46]. In this study, the distinction between the tegmen and testa was not observed, and the tegmen and endotesta may be fused based on the discovery of the tegmen layer in the testa. The exotesta, evaluated through SEM, was a relatively rigid cellular structure, indicating that it acts as a physical barrier. According to Corner’s [47] classification, which is based on such physical features, these taxa have exotestal seeds.

Protrusions are hypothesized to arise from the easy separation of attached parts in relation to physical force. Protrusions are distributed on the surface of the stamen where pollen develops in many taxa with wind pollination, requires dispersion by small force [48]. Accordingly, granulates may be a strategy for seed dispersion; however, in the genus *Mentzelia* of Loasaceae, there is evidence that they function in securing moisture for germination rather than in seed dispersion [49]. In the genus *Allium*, including *Allium cepa*, it is important to store sufficient moisture on the seed surface [50]. In summary, granulates in *Allium* seeds may be an evolutionary strategy to retain moisture for seed germination and expand the surface area, rather than a strategy for seed dispersion. The moisture absorption rate for seed germination in *A. koreanum* H.J.Choi & B.U.Oh (Subg. *Cepa*) exceeds 100% in less than 24 h [51]. *A. fistulosum* L. (Subg. *Cepa*) has a moisture content of over 80% after 5 h at 40 °C [52]. Some taxa in the genus *Allium* exhibit physiological or morphological dormancy [53]; after breaking dormancy, the taxa with protrusions have a high germination rate of over 95%, whereas *A. microdictyon,* in which protrusions were not confirmed, the germination rate is 17.9% (significantly lower than in other taxa) [54].

The results of this study, based on analyses of morphological characters, support the *Allium* classification in previous studies. The subgenus *Anguinum*, which includes *A. microdictyon* and *A. ulleungense*, was clearly distinct from other taxa, consistent with the molecular phylogenetic trees reported by Li et al. [55] and Choi et al. [56] indicating that this subgenus branched earlier than other subgenera. Choi et al. [56] analyzed morphological characters of flowers and leaves, demonstrating that a major splitting event occurred between subg. *Anguinum* and other subgenera. The absence of protrusions in the periclinal walls of *A. microdictyon* and *A. ulleungense*, despite their presence in more basal subgenera, indicates that these traits may have evolved after the divergence of *Anguinum*. The genera *Anguinum*, *Butomissa*, and *Rhizirideum* (including *A. anisopodium*) share substantial similarity in morphological characteristics and formed a group in the molecular phylogenetic tree; these findings indicate that the morphological characteristics can sufficiently explain the genetic relationships between taxa.

**Figure 9:**
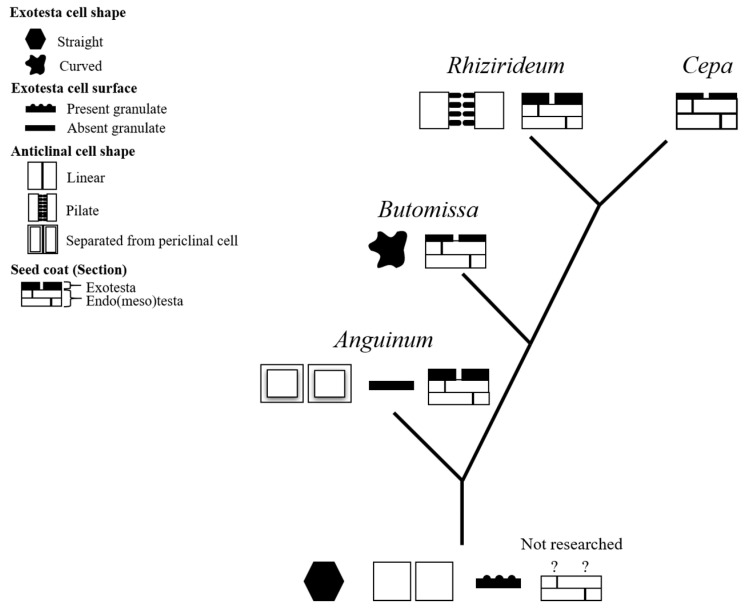
Phylogenetic tree of the morphological evolution of *Allium* seeds. The ancestral characters were based on the characters described in Choi et al. [56], and the ? marked cross-section of the seed coat was not described.

These analyses revealed that *A. anisopodium* in subg. *Rhizirideum* is closely related to *A. dumebuchum* in the same subgenus. Based on these micro-characteristics, the evolution of the seed coat can be described as follows (Figure 9).

(A) The protrusions on the seed coat basilar wall degenerated during the separation of the species from *Anguinum* and other subgenera.
(B) After the divergence of subg. *Butomissa*, the borders of the seed coat cells developed irregular shapes, and the thickness of the exotesta decreased.
(C) In subg. *Rhizirideum*, granulates formed between the intercellular anticlinal walls.
(D) In subg. *Cepa*, the thickness of the exotesta was reduced.

This study did not include all taxa in the genus *Allium*. Accordingly, the proposed evolutionary processes should be evaluated through morphological studies of additional taxa and a comprehensive phylogenetic analysis based on genetic traits.

In the phylogenetic tree presented in this study, *Rhizirideum* and *Cepa* formed two groups. The subg. *Rhizirideum* was divided into sect. *Tenuissima* and sect. *Rhizirideum*. Subgenus *Cepa* includes many taxa within *Allium* and contains several sections in the lower ranks. The section included in this study were sect. *sacculiferum*; however, there were no significant taxonomic differences in the phylogenetic analysis, suggesting that the seed traits are useful for classification at the subgenus level.

*Greenbelt* and *Cheongrim-buchu* are commercially available cultivars, sold as Chinese chives in the market. The CWRs of Chinese chives are *A. tuberosum* (primary) and *A. ramosum* L., *A. scabriscapum* Boiss. & Kotschy (secondary) [57,58]. According to the results of this study, although the both cultivars are derived from *A. tuberosum*, their morphological characteristics are similar to those of *A. sacculiferum* and *A. thumbergii* var. *teretifolium* in the same subgenus (Figure 8). It is possible that the morphological characteristics of *A. sacculiferum* or *A. thumbergii* var. *teretifolium* were acquired from *A. tuberosum* or from *A. sacculiferum* during breeding. For example, crop breeding has been conducted based on the results of Chuda & Adamus [59] which showed that *A. fistulosum* is resistant to biotic stress. *Dume-buchu*, developed from *A. dumebuchum*, had very similar micro-characteristics to those of *A. dumebuchum* in this study. However, observed differences in external characteristics may be due to selection or breeding with other wild species or cultivars.

## 4. Materials and Methods

### 4.1. Samples

Wild seed samples of *Allium* were obtained from the seed bank of the Baekdudaegan National Arboretum (BDNA) (Table 4). The seeds stored in the seed bank were collected directly from the wild and identified through purification and quality control; the collection number, collection date, collector, inspection date, and storage date after moisture measurement were recorded. Seeds of commercially available cultivars were purchased from seed sellers registered in Korea.

### 4.2. Analysis of External Morphological Characteristics

To measure the external morphological characteristics of *Allium* seeds, spectral images were obtained using a spectral image analyzer (VideometerLab 4, Videometer, Herlev, Denmark). After images were obtained using 19 wavelengths, the seeds and other parts were separated, and an nCDA (normalized Canonical Discriminant Analysis) file was created through an automatic statistical analysis using the wavelength information. After segmentation according to the level of nCDA activity, seeds were extracted and aligned using the corresponding segmentation. Each image was further analyzed to obtain basic seed information, such as length, width, and area, and additional information, such as beta shape a. For color, the Lab values calculated through image analysis were converted to sRGB through the H_65_ standard basic conversion process according to the standard of IEC 61966-2-1:1999 [60] and then compared with the RHS chart. The height of the seeds was measured using Vernier calipers (Traceable^®^, Webster, TX, USA).

#### Outline Analysis

To analyze the outline, images of the *Allium* seeds were obtained along the *x*-axis, *y*-axis, and *z*-axis using a stereo microscope (DVM6, Leica, Wetzlar, Germany). All *Allium* seeds were photographed using the z-stack function in the stereomicroscope shooting program Las X (Leica, Wetzlar, Germany) with all sides in focus. The images were converted to black and white through binarization according to the image color using the Python-based Colaboratory (Google Inc., USA, https://colab.research.google.com) program, and the outline was extracted through a contour analysis. The extracted outline was approximated and expressed as more than 200 points, and the coordinate values of the points on the image were extracted. The outline coordinate values are expressed in the form of *x*-, *y*-axis based on the pixel values on the image, and the coordinate values were separated into a separate file for each object and then used for analysis after error verification using the Momocs package version 1.4.1 in R [61]. The verified coordinate values were analyzed using PCA after Fourier transformation using the transform function in the Momocs package. eFourier is a simple transform that uses the difference between the *x*-axis and *y*-axis [62]. rFourier uses the radius of the sample and the cosine and sine functions, while tFourier uses the tangent function [63]. sFourier uses the values for 64 radii from the center of gravity [64].

### 4.3. Micromorphological Analysis

To confirm the micromorphological characteristics of the seeds, a scanning electron microscope (SEM) preprocessing process was used. Preprocessing involved fixing the seeds in a formalin acid alcohol solution (FAA) for at least 24 h, followed by incubation in 100% ethanol through an ethanol series and then drying the seeds using a CPD (Critical Point Dryer; Samdri-PVT-3D, Tousimis, Rockville, MD, USA). The dried seeds were placed on aluminum stubs with carbon tape attached and then coated with Au at 3 mA for 300 s using an ion coater (SPT-20, COXEM, Daejeon, Republic of Korea). Images were obtained using SEM (CX-200, COXEM, Daejeon, Republic of Korea) at an acceleration voltage of 15 kV and focal length of 10 ± 5 mm.

The microscopic characteristics of the seeds evaluated in the study included the shape of the periclinal and anticlinal walls composing the seed coat and the presence or absence of protrusions. In addition, images were taken at high magnification to measure the size of the protrusions. Based on images of various parts of a single sample, the size was calculated using image accumulation. Images of the boundaries and groups of cells composing the seed coats were obtained to confirm the shapes of the periclinal and anticlinal walls. The shapes of the boundaries of each cell in each image were classified according to the criteria of Hickey & King [65].

### 4.4. Analysis of Phylogenetic Relationships

Phylogenetic analyses of cultivars and wild taxa were performed based on two datasets: (a) outlines and (b) external and micromorphological trait data. The initial analysis based on using outlines was conducted to verify the utility of quantitative variables for taxon discrimination.

In the analysis of taxa using outlines alone, the optimal Fourier transform values for each axis of the seeds were used. The optimal Fourier values were determined according to the degree of explanation and discrimination ability of each variable using each PCA value, and the transformed Fourier values were used for phylogenetic analysis. The outlines of all seeds for each group of each taxon were extracted using the MSHAPE function of the Momocs package, and a comprehensive phylogenetic analysis was performed using the average values.

For phylogenetic analyses, both quantitative and qualitative variables were transformed. Quantitative variables, were normalized to address variation in the weights of each variable, while qualitative variables, were factorized for common traits. The transformed variables were subjected to FAMD (Factor Analysis Mixed Data) using the FAMD function of the FactoMineR package version 2.11 [66] and factoextra package version 1.0.7.

In the phylogenetic analysis using the outline and the comprehensive analysis using all variables, distances were calculated for each variable. In particular, Gower distances were calculated using the Cluster package in the R program, and other distances, such as Manhattan distances, were calculated using the stats and ade4 packages version 1.7-23 [67]. Phylogenies were inferred using fastME, NJ, and BNJ distance algorithms of the ape package version 5.8-1 [68], UNJ of phangorn version 2.12.1 [69], and hclust of stats. Bootstrap values were calculated using the boot.phylo function of the ape package. The likelihood value for the phylogenetic tree based on each calculated distance matrix was determined using the motmot package version 2.1.3 [70], and the phylogenetic tree with the optimal value was selected as the final phylogenetic tree.

## 5. Conclusions

CWR studies focused on cultivars and wild taxa distributed in Korea are lacking. The *Allium* taxa in this study have not been reported in CROPTRUST or the USDA. Therefore, we suggest the inclusion *A. saccuiferum* and *A. thumbergii* var. *teretifolium* within subg. *Cepa* in both Korean and international CWR inventories, as supported by our observation that these taxa are phylogenetically very closely related to *A. tuberosum*, categorized as a primary CWR, and share numerous morphological characteristics.

Allium species, including cultivated varieties, show high morphological similarity. We identified quantitative morphological parameters, particularly the shape outline along the *x*-axis and micromorphological traits, able to effectively distinguish taxa at the subgenus level. Moreover, wild taxa harboring valuable genetic resources and cultivated varieties exhibited highly similar morphological features. Although there is strong potential for developing new cultivars through the utilization of novel genetic traits from wild relatives, these findings also highlight the challenge of morphological differentiation between cultivated and wild taxa, posing a potential threat to the conservation of wild taxa. We emphasize the importance of ongoing taxonomic and phylogenetic analyses to update and refine the classification within CWR inventories. We expect our findings to contribute to the improved conservation and utilization of CWRs.

## Figures and Tables

**Table 1:** *t*-test after F-test result of traits among groups in two species of *Allium*, *A. longistylum* and *A. anisopodium*. H is seed height, Beta is Beta shape, Rad Avg is Phi Rad Statistic Average, Comp Eclipse is ellipse compactness. ** *p* < 0.01, * *p* < 0.05, - *p* > 0.05.

	H	Beta		Rec	Skew		Kurt		Rad Avg	Point Ness	Comp Eclipse
		a	b		x	y	x	y			
*A. longistylum* 1–2	-	-	-	-	-	-	-	-	-	-	-
*A. longistylum* 2–3	-	-	-	-	-	-	-	-	-	-	-
*A. longistylum* 1–3	-	-	-	-	-	-	-	-	-	-	-
*A. anisopodium* 1–2	-	-	**	-	-	-	*	*	-	-	**

**Table 2:** Result of analysis of variance (ANOVA) test about seed each axis outlines. *** *p* < 0.001.

*x*-axis	df	Wilks	Approx. F	Num Df	Den Df	Pr (>F)
taxa	13	0.058755	24.862	221	17505	<2.2 × 10^−16^ ***
Residuals	1699					
*y*-axis						
taxa	13	0.071127	32.882	156	14811	<2.2 × 10^−16^ ***
Residuals	1675					
*z*-axis						
taxa	13	0.25803	4.7473	507	20763	<2.2 × 10^−16^ ***
Residuals	1715					

**Table 3:** Comparison of granulate lengths among taxa. Lengths were calculated from SEM images. The same lowercase letters indicate a significance length difference.

Taxa	Length	Taxon	Length
*A. tuberosum*	^ce^ 1.01 ± 0.25	*A. thunbergii*	^e^ 1.08 ± 0.22
*A. spirale*	^bc^ 0.95 ± 0.18	*A. thunbergii* var. *teretifolium*	^f^ 1.31 ± 0.34
*A. sacculiferum*	^a^ 0.79 ± 0.18	*A. anisopodium*	^a^ 0.71 ± 0.17
*A. taquetii*	^bc^ 0.95 ± 0.20	*A. ulleungense*	Absent
*A. dumebuchum*	^a^ 0.72 ± 0.15	*A. microdictyon*	Absent
*A. longistylum* 1	^a^ 0.76 ± 0.21	*Chungrim-buchu*	^ce^ 1.04 ± 0.27
*A. longistylum* 2	^b^ 0.91 ± 0.20	*Dume-buchu*	^bc^ 0.95 ± 0.23
*A. longistylum* 3	^bc^ 0.93 ± 0.22	*Greenbelt*	^g^ 1.44 ± 0.34

**Table 4:** List of seed samples and vouchers. For this study, over 100 seeds were used from each voucher.

Species	Bank No.	Date	Region
*Allium anisopodium* Ledeb.	2021-006804	14 November 2021	Okdo-myeon, Gunsan-si, Jeollabuk-do, Korea Republic
*A. dumebuchum* H.J.Choi	2021-006822	2021	Subi-myeon, Yeongyang-gun, Gyeongsangbuk-do, Korea Republic
*A. longistylum* Baker	2021-006773	3 November 2021	Galmal-eup, Cheolwon-gun, Gangwon-do, Korea Republic
	2021-006832	25 October 2021	Dongsong-eup, Cheolwon-gun, Gangwon-do, Korea Republic
	2021-006833	25 October 2021	Gunnam-myeon, Yeoncheon-gun, Gyeonggi-do, Korea Republic
*A. microdictyon* Prokh.	2021-006513	2021	Punggi-eup, Yeongju-si, Gyeongsangbuk-do, Korea Republic
*A. sacculiferum* Maxim.	2019-008952	9 October 2019	Gogae-myeon, Geochang-gun, Gyeongsangnam-do, Korea Republic
*A. spirale* Willd.	2021-006539	2 November 2021	Gangmun-dong, Gangneung-si, Gangwon-do, Korea Republic
*A. taquetii* H.Lév. & Vaniot	2021-006802	31 October 2021	Saekdal-dong, Seogyipo-si, Jeju-do, Korea Republic
*A. thunbergii* G.Don	2020-040355	14 October 2020	Ganseong-eup, Gosung-gun, Gangwon-do, Korea Republic
*A. thunbergii* var. *teretifolium* H.J.Choi & B.U.Oh	2018-012360	22 October 2018	Macheon-myeon, Hamyang-gun, Gyeongsangnam-do, Korea Republic
*A. tuberosum* Rottler ex Spreng.	2021-006775	1 October 2021	Shindong-eup, Jeongseon-gun, Gangwon-do, Korea Republic
*A. ulleungense* H.J.Choi & N.Friesen	2021-005218	22 June 2021	Ulleung-eup, Ulleung-gun, Gyeongsangbuk-do, Korea Republic

## Data Availability

Data are contained within the article.

## Abbreviations

CWR	Crop Wild Relative
FAMD	Factor Analysis Mixed Data
PCA	Principal Component Analysis
SEM	Scanning Electron Microscope

## Appendix A

The table shows measured data for each *Allium* taxon. R, G, and B values were transformed from Lab values. Betashape a and b is value of ellipse shape characterizaion bolow (betashape a) and top (betashape b). The value is from 1 (rectangular shape) to over 2 (pointed shape) range. Circle or Eclipse Compactness is the maximum ratio between seed outline and circle or ellipse. W/A is the Width/Area ratio, W/L is the Width/Length ratio. Rectangularity is the maximum ratio of the fit rectangle. Fit rectangle is constructed from the maximum Length and Width of the seed. Region Horizonal Length Mean is the average all of seed lengths, and Region Vertical Length Mean is the average all of seed widths. PhiRad Symmetry shows the radians between the outline point and next outline point, and the center of angles is the seed center. PhiRad Statistics is similar to PhiRad Symmetry; it is length of the seed center–outline point. Moments X and Y are spatial centers of the seed *x*- and *y*-axes. Pointness is calculated as (2 × wide area by length/2)/area.

**Table A1:** External morphology (R, G, B, legnth, width, height and betashape) of *Allium* seeds. Averages ± standard deviations for each taxon are shown.

Taxon	R	G	B	Length (mm)	Width (mm)	Height (mm)	Betashape a	Betashape b
*Allium longistylum* 1	48.02 ± 5.11	42.99 ± 5.37	33.63 ± 6.58	2.64 ± 0.31	1.87 ± 0.21	0.49 ± 0.46	1.57 ± 0.24	1.44 ± 0.17
*A. longistylum* 2	42.61 ± 4.27	38.74 ± 4.42	29.68 ± 5.64	3.08 ± 0.30	2.06 ± 0.21	0.55 ± 0.13	1.55 ± 0.17	1.45 ± 0.16
*A. longistylum* 3	50.44 ± 7.06	47.69 ± 7.30	41.17 ± 8.26	3.18 ± 0.37	2.08 ± 0.23	0.49 ± 0.15	1.53 ± 0.20	1.41 ± 0.14
*A. tuberosum*	48.35 ± 4.36	46.78 ± 5.05	38.72 ± 6.54	3.25 ± 0.19	2.25 ± 0.13	1.36 ± 0.17	1.66 ± 0.15	1.54 ± 0.12
*A. dumebuchum*	56.59 ± 5.04	54.23 ± 4.94	48.42 ± 5.19	3.39 ± 0.34	2.13 ± 0.19	0.73 ± 0.17	1.59 ± 0.15	1.44 ± 0.13
*A. anisopodium*	55.97 ± 5.61	51.67 ± 5.78	43.35 ± 6.30	2.69 ± 0.28	2.09 ± 0.23	0.82 ± 0.24	1.52 ± 0.18	1.34 ± 0.13
*A. taquaii*	32.81 ± 6.32	29.60 ± 6.33	17.26 ± 8.57	3.03 ± 0.22	2.59 ± 0.27	2.22 ± 0.31	1.58 ± 0.13	1.50 ± 0.10
*A. microdictyon*	45.55 ± 6.83	42.03 ± 6.60	33.93 ± 8.01	3.24 ± 0.27	2.27 ± 0.29	0.73 ± 0.51	1.54 ± 0.16	1.44 ± 0.13
*A. ulleungense*	48.23 ± 4.66	44.08 ± 5.05	35.80 ± 5.98	2.36 ± 0.41	1.77 ± 0.23	1.60 ± 0.47	1.80 ± 0.33	1.59 ± 0.22
*A. thunbergii*	47.37 ± 4.23	42.84 ± 4.40	33.61 ± 5.46	2.35 ± 0.28	1.77 ± 0.24	1.38 ± 0.59	1.76 ± 0.36	1.53 ± 0.17
*var. terafolium*	47.54 ± 4.53	44.89 ± 5.12	37.93 ± 6.09	3.44 ± 0.35	2.11 ± 0.18	1.50 ± 1.12	1.58 ± 0.13	1.44 ± 0.10
*A. saccifolium*	36.13 ± 7.05	33.46 ± 7.45	22.88 ± 9.60	3.03 ± 0.21	2.51 ± 0.36	1.99 ± 0.81	1.60 ± 0.14	1.51 ± 0.10
*A. spirale*	51.02 ± 4.71	48.68 ± 5.05	40.95 ± 5.93	2.91 ± 0.38	1.85 ± 0.37	1.33 ± 0.34	1.52 ± 0.16	1.40 ± 0.12
*Chungrim-buchu*	48.47 ± 4.21	44.89 ± 4.26	35.52 ± 4.75	3.59 ± 0.24	2.53 ± 0.23	1.12 ± 0.42	1.61 ± 0.13	1.44 ± 0.11
*Greenbelt*	54.24 ± 5.17	50.92 ± 5.20	43.46 ± 5.78	3.15 ± 0.36	2.18 ± 0.28	0.94 ± 0.47	1.55 ± 0.12	1.44 ± 0.08
*Dume-buchu*	48.44 ± 5.42	45.19 ± 5.68	36.12 ± 6.86	3.18 ± 0.24	2.31 ± 0.23	1.43 ± 0.25	1.55 ± 0.10	1.42 ± 0.08

**Table A2:** External morphology (compactness, area, W/A. W/L, rectangularity and rigion length mean) of *Allium* seeds. Averages ± standard deviations for each taxon are shown.

Taxon	Ellipse Compactness	Circle Compactness	Area (mm^2^)	W/A	W/L	Rectangularity	Region Horizonal Length Mean	Region Vertical Length Mean
*Allium longistylum* 1	0.98 ± 0.01	0.69 ± 0.08	3.67 ± 0.78	0.52 ± 0.08	0.71 ± 0.08	0.78 ± 0.03	0.73 ± 0.05	0.74 ± 0.05
*A. longistylum* 2	0.99 ± 0.01	0.66 ± 0.08	4.77 ± 0.80	0.45 ± 0.07	0.67 ± 0.08	0.78 ± 0.03	0.74 ± 0.03	0.76 ± 0.03
*A. longistylum* 3	0.98 ± 0.01	0.65 ± 0.08	4.98 ± 0.95	0.66 ± 0.08	0.78 ± 0.03	0.78 ± 0.03	0.74 ± 0.04	0.76 ± 0.04
*A. tuberosum*	0.99 ± 0.01	0.70 ± 0.05	5.37 ± 0.42	0.42 ± 0.02	0.69 ± 0.05	0.76 ± 0.02	0.73 ± 0.03	0.74 ± 0.03
*A. dumebuchum*	0.99 ± 0.01	0.62 ± 0.07	5.37 ± 0.86	0.40 ± 0.05	0.63 ± 0.07	0.77 ± 0.02	0.74 ± 0.03	0.75 ± 0.03
*A. anisopodium*	0.97 ± 0.02	0.74 ± 0.08	4.13 ± 0.78	0.51 ± 0.06	0.78 ± 0.07	0.77 ± 0.03	0.73 ± 0.04	0.74 ± 0.04
*A. taquaii*	0.99 ± 0.02	0.86 ± 0.07	5.89 ± 0.93	0.44 ± 0.03	0.85 ± 0.07	0.79 ± 0.02	0.75 ± 0.02	0.75 ± 0.02
*A. microdictyon*	0.99 ± 0.01	0.69 ± 0.11	5.49 ± 0.85	0.42 ± 0.04	0.70 ± 0.10	0.78 ± 0.02	0.74 ± 0.03	0.75 ± 0.03
*A. ulleungense*	0.97 ± 0.02	0.74 ± 0.09	2.98 ± 0.96	0.62 ± 0.11	0.76 ± 0.09	0.77 ± 0.03	0.70 ± 0.05	0.71 ± 0.05
*A. thunbergii*	0.98 ± 0.01	0.75 ± 0.11	2.99 ± 0.57	0.60 ± 0.07	0.76 ± 0.11	0.78 ± 0.03	0.71 ± 0.04	0.72 ± 0.05
*var. terafolium*	0.98 ± 0.01	0.61 ± 0.07	5.45 ± 0.85	0.39 ± 0.05	0.62 ± 0.07	0.77 ± 0.02	0.74 ± 0.03	0.75 ± 0.03
*A. saccifolium*	0.99 ± 0.01	0.83 ± 0.11	5.69 ± 1.04	0.44 ± 0.03	0.83 ± 0.11	0.79 ± 0.02	0.74 ± 0.02	0.75 ± 0.03
*A. spirale*	0.98 ± 0.01	0.62 ± 0.08	4.12 ± 1.35	0.47 ± 0.06	0.63 ± 0.07	0.78 ± 0.03	0.74 ± 0.03	0.76 ± 0.03
*Chungrim-buchu*	0.97 ± 0.01	0.69 ± 0.08	6.71 ± 0.83	0.38 ± 0.03	0.71 ± 0.07	0.76 ± 0.02	0.73 ± 0.03	0.74 ± 0.03
*Greenbelt*	0.98 ± 0.01	0.68 ± 0.05	5.26 ± 1.16	0.43 ± 0.05	0.70 ± 0.05	0.78 ± 0.02	0.75 ± 0.03	0.76 ± 0.03
*Dume-buchu*	0.98 ± 0.01	0.72 ± 0.07	5.58 ± 0.8	0.42 ± 0.03	0.73 ± 0.07	0.78 ± 0.02	0.75 ± 0.02	0.98 ± 0.01

**Table A3:** External morphology (skew, phirad symmetry, kurt and moment) of *Allium* seeds. Averages ± standard deviations for each taxon are shown.

Taxon	Skew X	Skew Y	PhiRad Symmetry Stat Mean	PhiRad Symmetry Stat Max	Kurt X	Kurt Y	Moment X	Moment Y
*Allium longistylum* 1	0.08 ± 0.06	0.07 ± 0.06	0.02 ± 0.01	0.05 ± 0.02	0.99 ± 0.06	0.99 ± 0.07	232.07 ± 52.67	476.74 ± 116.86
*A. longistylum* 2	0.07 ± 0.05	0.05 ± 0.04	0.02 ± 0.01	0.04 ± 0.01	1.00 ± 0.04	1.00 ± 0.05	286.56 ± 58.17	645.25 ± 127.15
*A. longistylum* 3	0.08 ± 0.06	0.07 ± 0.05	0.02 ± 0.01	0.04 ± 0.01	1.00 ± 0.05	1.00 ± 0.06	293.26 ± 65.48	692.61 ± 157.83
*A. tuberosum*	0.09 ± 0.03	0.06 ± 0.04	0.02 ± 0.00	0.05 ± 0.01	0.98 ± 0.04	0.96 ± 0.04	343.56 ± 35.91	678.81 ± 70.79
*A. dumebuchum*	0.08 ± 0.05	0.08 ± 0.04	0.02 ± 0.01	0.04 ± 0.01	0.99 ± 0.04	0.99 ± 0.05	303.82 ± 55.43	772.70 ± 151.24
*A. anisopodium*	0.13 ± 0.05	0.10 ± 0.07	0.02 ± 0.01	0.05 ± 0.01	0.99 ± 0.06	1.01 ± 0.06	288.06 ± 63.20	499.20 ± 96.53
*A. taquaii*	0.04 ± 0.04	0.04 ± 0.03	0.01 ± 0.01	0.03 ± 0.01	0.99 ± 0.03	0.99 ± 0.04	455.05 ± 91.23	608.27 ± 80.86
*A. microdictyon*	0.09 ± 0.06	0.06 ± 0.04	0.02 ± 0.01	0.04 ± 0.02	1.00 ± 0.04	1.00 ± 0.05	349.03 ± 89.14	710.96 ± 122.01
*A. ulleungense*	0.11 ± 0.08	0.09 ± 0.08	0.02 ± 0.01	0.04 ± 0.01	0.94 ± 0.07	0.93 ± 0.09	203.53 ± 56.48	366.94 ± 149.44
*A. thunbergii*	0.11 ± 0.06	0.10 ± 0.08	0.02 ± 0.01	0.04 ± 0.01	0.95 ± 0.07	0.94 ± 0.09	207.55 ± 51.41	358.65 ± 78.80
*var. terafolium*	0.11 ± 0.04	0.07 ± 0.05	0.02 ± 0.01	0.05 ± 0.01	1.00 ± 0.03	0.99 ± 0.04	303.28 ± 50.46	801.30 ± 157.57
*A. saccifolium*	0.04 ± 0.03	0.04 ± 0.04	0.01 ± 0.01	0.03 ± 0.01	0.98 ± 0.03	0.98 ± 0.04	428.57 ± 111.48	608.46 ± 80.55
*A. spirale*	0.08 ± 0.05	0.06 ± 0.04	0.02 ± 0.01	0.05 ± 0.02	1.00 ± 0.04	1.01 ± 0.05	240.26 ± 102.68	580.16 ± 155.20
*Chungrim-buchu*	0.16 ± 0.05	0.09 ± 0.05	0.02 ± 0.01	0.05 ± 0.01	0.98 ± 0.04	0.98 ± 0.04	431.67 ± 78.63	869.77 ± 112.08
*Greenbelt*	0.13 ± 0.04	0.05 ± 0.04	0.02 ± 0.01	0.04 ± 0.01	1.00 ± 0.03	1.00 ± 0.03	329.77 ± 82.32	683.26 ± 152.77
*Dume-buchu*	0.15 ± 0.04	0.07 ± 0.04	0.02 ± 0.01	0.04 ± 0.01	1.00 ± 0.03	1.00 ± 0.03	370.98 ± 73.26	691.09 ± 99.41

**Table A4:** External morphology (moment ratio, perimeter, phirad statistics and pointness) of *Allium* seeds. Averages ± standard deviations for each taxon are shown.

Taxon	Moment Y Ratio	Perimeter (mm)	PhiRad Statistics Avg	PhiRad Statistics Min	PhiRad Statistics Max	Pointness
*Allium longistylum* 1	0.70 ± 0.08	6.65 ± 0.72	0.48 ± 0.02	0.41 ± 0.03	0.54 ± 0.02	0.08 ± 0.05
*A. longistylum* 2	0.67 ± 0.08	7.59 ± 0.64	0.48 ± 0.01	0.43 ± 0.02	0.54 ± 0.02	0.07 ± 0.04
*A. longistylum* 3	0.66 ± 0.08	7.82 ± 0.77	0.48 ± 0.01	0.42 ± 0.03	0.54 ± 0.02	0.08 ± 0.05
*A. tuberosum*	0.71 ± 0.05	7.95 ± 0.36	0.48 ± 0.01	0.43 ± 0.02	0.53 ± 0.01	0.09 ± 0.04
*A. dumebuchum*	0.63 ± 0.07	8.06 ± 0.69	0.48 ± 0.01	0.42 ± 0.03	0.54 ± 0.02	0.10 ± 0.04
*A. anisopodium*	0.76 ± 0.07	7.09 ± 0.64	0.48 ± 0.01	0.40 ± 0.03	0.56 ± 0.02	0.11 ± 0.06
*A. taquaii*	0.86 ± 0.07	7.98 ± 0.62	0.48 ± 0.01	0.44 ± 0.03	0.52 ± 0.02	0.06 ± 0.03
*A. microdictyon*	0.70 ± 0.10	8.03 ± 0.64	0.48 ± 0.01	0.42 ± 0.02	0.53 ± 0.02	0.07 ± 0.03
*A. ulleungense*	0.76 ± 0.09	5.85 ± 0.97	0.46 ± 0.02	0.40 ± 0.03	0.54 ± 0.02	0.11 ± 0.07
*A. thunbergii*	0.76 ± 0.10	5.83 ± 0.59	0.47 ± 0.01	0.41 ± 0.03	0.54 ± 0.02	0.12 ± 0.07
*var. terafolium*	0.62 ± 0.07	8.18 ± 0.69	0.48 ± 0.01	0.42 ± 0.02	0.55 ± 0.02	0.09 ± 0.05
*A. saccifolium*	0.83 ± 0.11	7.89 ± 0.71	0.48 ± 0.01	0.44 ± 0.02	0.52 ± 0.02	0.06 ± 0.04
*A. spirale*	0.63 ± 0.08	7.04 ± 0.99	0.48 ± 0.01	0.42 ± 0.03	0.54 ± 0.02	0.08 ± 0.04
*Chungrim-buchu*	0.71 ± 0.07	8.96 ± 0.52	0.48 ± 0.01	0.41 ± 0.02	0.55 ± 0.02	0.09 ± 0.05
*Greenbelt*	0.70 ± 0.05	7.83 ± 0.89	0.48 ± 0.01	0.43 ± 0.02	0.55 ± 0.02	0.07 ± 0.04
*Dume-buchu*	0.73 ± 0.07	8.09 ± 0.59	0.49 ± 0.01	0.43 ± 0.02	0.55 ± 0.02	0.08 ± 0.04
